# Theoretical and computational study of a quad-band optical switch and three-bit encoder based on a graphene metasurface

**DOI:** 10.1016/j.isci.2025.114524

**Published:** 2025-12-23

**Authors:** Jiacheng Dai, Jun Zhu

**Affiliations:** 1 School of Electronic and Information Engineering/School of Integrated Circuits, Guangxi Normal University, Guilin 541004, China; 2 Key Laboratory of Integrated Circuits and Microsystems (Guangxi Normal University), Education Department of Guangxi Zhuang Autonomous Region, Guilin 541004, China

**Keywords:** physics, optics, applied sciences

## Abstract

To address the issues of insufficient tunability and high losses in existing metamaterial devices, this study proposes a tunable metamaterial device based on graphene. Coupling effects among these units under normal-incidence x-polarized terahertz irradiation result in the emergence of four transmission dips and three electromagnetically induced transparency resonances, the latter achieving transmittance values above 90%. Under Fermi level adjustment in graphene, the device exhibits excellent quad-band optical switching performance, achieving a maximum modulation depth of 92.1%, alongside an insertion loss of only 0.01 dB alongside an 11 dB extinction ratio. Furthermore, the proposed device can also realize 3-bit encoding functions at 3.18, 4.8, and 6.38 THz. The structure demonstrates remarkable slow-light tunability, with the group refractive index peaking at 623. This research provides a novel device design approach for terahertz communications, wide-field imaging, and multifunctional sensing applications.

## Highlights

• Realized a high-performance quad-band optical switch using graphene metamaterials
• Enables 3-bit encoding with strong modulation depth and minimal insertion loss
• Features tunable slow-light effect and wide-angle incidence insensitivity

## Graphical abstract

**Figure:**
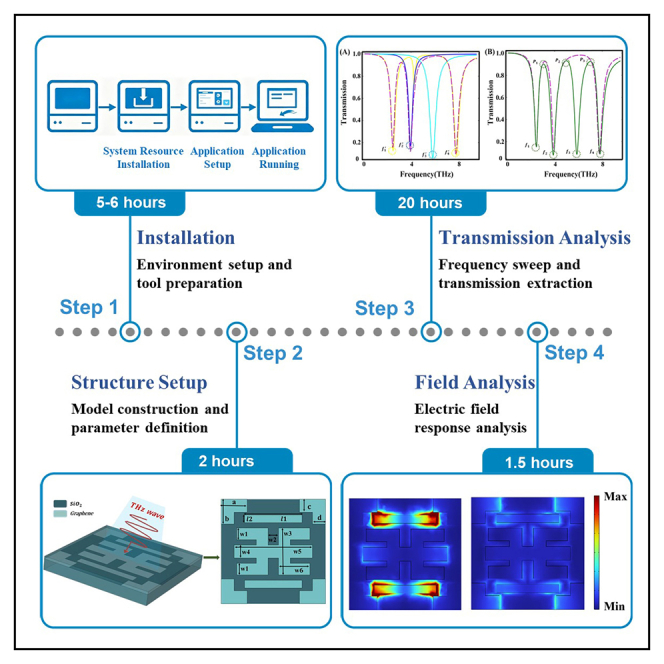

## Introduction

Electromagnetically induced transparency (EIT) is a quantum interference phenomenon that enables strong suppression of absorption and enhancement of transmission through coherent coupling between light and the medium.^1^^,^^2^^,^^3^^,^^4^ In recent years, researchers have achieved a quasi-EIT effect in the microwave and terahertz bands through bright-dark mode coupling in metamaterials.^5^^,^^6^^,^^7^^,^^8^ However, existing metamaterial devices still face challenges such as poor tunability, high losses, and low sensitivity, which limit their applications in communication and sensing.

In recent years, graphene-based metamaterials have demonstrated significant research value in fields, such as terahertz communication, holographic imaging, optoelectronic devices, high-sensitivity biosensing, and perfect absorbers, owing to their excellent tunability and strong field-localization capabilities.^9^^,^^10^^,^^11^^,^^12^^,^^13^^,^^14^^,^^15^^,^^16^ In 2021, Kumar et al. introduced a graphene metasurface that, when the Fermi level was tuned to 1.0 eV, exhibited a group delay of 0.76 ps and a group refractive index of 22.78, demonstrating its potential for slow-light modulation.^17^ In 2022, Gong et al. designed a dual-functional metasurface modulator that realized a 92% amplitude-modulation depth (MD) at 0.55 THz.^18^ In 2023, Deng et al. designed a dynamically tunable graphene metal-insulator phase transition metasurface that used dual metal-insulator phase transitions of different frequencies and intensities to achieve encrypted imaging.^19^ In 2024, Nie et al. proposed a graphene-based horizontal-strip-vertical dual-resonator metasurface structure that, by tuning the Fermi level, achieved a three-frequency asynchronous optical switching function.^20^ In 2025, Cui et al. designed a dynamically tunable, graphene-based, co-ordinated triple-band terahertz perfect absorber that achieved ultrahigh absorption rates of 99.9%, 99.8%, and 98.3%, and exhibited polarization-insensitive, wide-angle absorption characteristics.^21^ These studies have advanced the engineering applications of graphene metamaterials in terahertz functional devices, providing key technological support for performance breakthroughs in communication, imaging, and sensing systems.^22^^,^^23^^,^^24^^,^^25^^,^^26^^,^^27^

This study proposes a graphene-based tunable metamaterial device that achieves multi-band EIT dynamic modulation in the terahertz region. The device is designed with an integrated composite resonant system composed of dual rectangular resonators, L-shaped resonator, and a trident-shaped resonator. The high carrier mobility and Fermi level of graphene provide a solution to overcome the static response limitations of traditional metallic metamaterials.^28^^,^^29^ Through gate voltage variation, flexible control over the EIT resonance frequency and MD can be achieved. Theoretical simulations indicate that the device exhibits quad-band optical switching functionality within the terahertz band. In addition, the designed device also features three-bit encoding capability, excellent slow-light characteristics, and outstanding incident-angle insensitivity across broad angular variations. It provides a multifunctional integrated platform for terahertz communication, biomedical imaging, environmental monitoring, and lays a theoretical and technological foundation for the design of next-generation intelligent photonic devices.

### Structural design and theoretical analysis

#### Structural design

**Figure 1:**
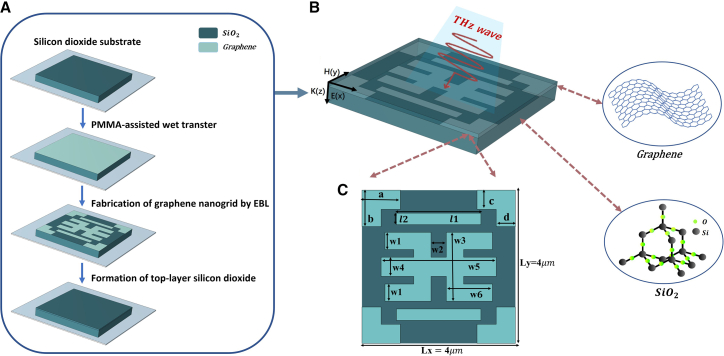
Graphene metamaterial fabrication and structure (A) Fabrication steps of the graphene metamaterial. (B) Three-dimensional model of the structure. (C) Top-down view of the metamaterial structure.

We develop a graphene-metamaterial hybrid structure that exhibits triple-band EIT. Figure 1 shows the fabrication steps of the graphene metamaterial. As illustrated in Figure 1B, the structure consists of metal electrodes, a double-layer silicon dioxide dielectric (with a relative permittivity of 3.9) and an intermediate graphene layer. The core functional layer features a symmetric composite graphene structure, consisting of two rectangular strips, four sets of L-shaped strips, and a centrally symmetric trident-shaped graphene element. According to Figure 1C, the geometric parameters of the two rectangular graphene strips are *l*1 = 2.2 μm and *l*2 = 0.3 μm; the geometric parameters of the L-shaped graphene strips are *a* = 1 μm, *b* = 0.95 μm, *c* = 0.5 μm, and *d* = 0.5 μm. The central graphene structure is defined by the following geometric parameters: *w*1 = 0.45 μm, *w*2 = 0.4 μm, *w*3 = 1.8 μm, *w*4 = 0.5 μm, *w*5 = 3 μm, and *w*6 = 1.2 μm. The proposed structure was numerically simulated in COMSOL. Multiphysics based on the finite element method (FEM). The overall structure is symmetrically and periodically distributed in the *x-y* plane, with lattice periods of *Lx* = *Ly* = 4 μm. The incident terahertz wave propagates along the negative *z*-direction, where port boundaries are defined, and a physics-controlled mesh is used for meshing.

For the proposed structure, high-mobility monolayer graphene is chosen as the functional material. Initially, the monolayer graphene was grown on copper foil by a standard chemical vapor deposition process.^30^ It was then transferred onto a SiO_2_ substrate using a polymethylmethacrylate-assisted wet transfer. Subsequently, electron beam lithography and plasma etching were performed, followed by encapsulation with an ion gel layer on the surface.^31^ In electromagnetic simulations, an infinite periodic array is simulated using Floquet conditions set on the *x-y* boundaries. The terahertz wave impinges perpendicularly along the *z*-direction and exhibits polarization along both the *x-* and *y*-orientations.

#### Theoretical analysis

In this design, monolayer graphene is modeled with an effective thickness of 0.335 nm, and its surface conductivity is determined by both interband and intraband transitions, as given by the Kubo formula^32^^,^^33^^,^^34^:

(Equation 1) $$\begin{array}{c} \sigma \left(\omega ,\mu_{c},\Gamma ,T\right)={\sigma_{inter}\left(\omega ,\mu_{c},\Gamma ,T\right)+\sigma }_{intra}\left(\omega ,\mu_{c},\Gamma ,T\right) \end{array}$$

(Equation 2) $$\begin{array}{c} \sigma_{inter}\left(\omega ,\mu_{c},\Gamma ,0\right)=-\frac{ie^{2}}{4\pi h}\ln\left[\frac{2\left|\mu_{c}-\left(\omega -i2\Gamma \right)h\right|}{2\left|\mu_{c}+\left(\omega -i2\Gamma \right)h\right|}\right] \end{array}$$

(Equation 3) $$\begin{array}{c} \sigma_{intra}\left(\omega ,\mu_{c},\Gamma ,T\right)=-\frac{ie^{2}k_{B}T}{\pi h\left(\omega -i2\Gamma \right)}\left[\frac{\mu_{c}}{k_{B}T}+\ln\left(e^{-\frac{\mu_{c}}{{Tk}_{B}}}+1\right)\right] \end{array}$$

where *e* is the elementary charge, *μ*_*c*_ denotes the chemical potential of graphene, *T* is the operating temperature, *h* represents Planck’s constant, *Γ* is the average number of Coulomb collisions experienced by charged particles per unit time, *k*_*B*_ is the Boltzmann constant, and *ω* denotes the angular frequency associated with the terahertz wave’s incidence.

The operating ambient temperature is set to *T* = 300*K*. At terahertz frequencies and under room temperature conditions, photon energy falls well short of the Fermi level. Consequently, graphene’s surface conductivity is mainly influenced by intraband transitions, with interband transitions considered negligible. Thus, the Kubo formula can be reduced to^35^

(Equation 4) $$\begin{array}{c} \sigma \left(\omega ,\mu_{c},\Gamma ,T\right)=N_{g}\frac{ie^{2}\mu_{c}}{\pi h^{2}\left(\omega +i2\Gamma \right)} \end{array}$$

here, *N*_*g*_ denotes how many graphene layers are incorporated in the structure. From the above equation, one can then determine the complex permittivity of graphene.

(Equation 5) $$\begin{array}{c} \varepsilon =1+\frac{i\sigma }{\left(\varepsilon_{0}\omega t\right)} \end{array}$$

here, *t* denotes the thickness of the graphene film, and *ε*_0_ = 8.854 × 10^−12^*F*/*m* is the vacuum permittivity.

In graphene, the imaginary part of the conductivity plays a crucial role in determining the propagation characteristics of surface plasmon polaritons (SPPs). When graphene exhibits a negative imaginary component in its conductivity (*Im*(*σ*_*g*_) < 0), graphene exhibits dielectric-like electromagnetic properties, and only *x*-polarized SPP modes are supported. In contrast, in scenarios where the imaginary part of the conductivity is greater than zero (*Im*(*σ*_*g*_) > 0), graphene shows metallic-like properties, and its metasurface supports only *y*-polarized modes. In this study, the condition with a positive imaginary conductivity is adopted, and the propagation constant *β* can be expressed as^36^:

(Equation 6) $$\begin{array}{c} \beta =k_{0}\sqrt{\varepsilon_{r}-{\left(\frac{2\varepsilon_{r}}{\eta_{0}\sigma_{g}}\right)}^{2}} \end{array}$$

here, *σ*_*g*_ represents the surface conductivity of graphene, *η*_0_ represents the free-space intrinsic impedance, *ε*_*r*_ is the silicon dioxide’s relative permittivity, and *k*_0_ = 2*π*/*λ* refers to the wave vector.

#### Analysis of simulation results

**Figure 2:**
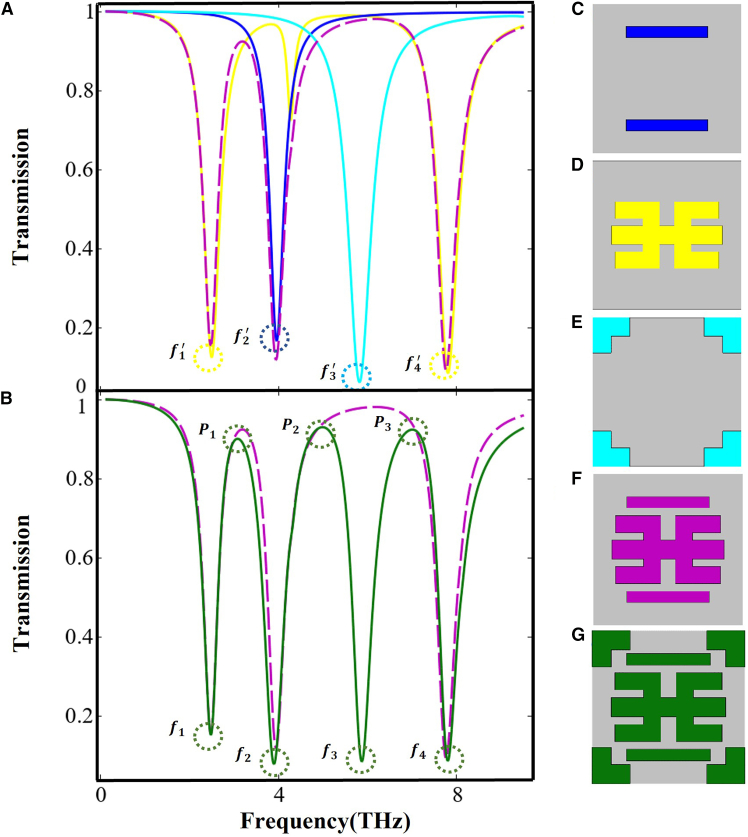
Transmission responses of graphene components and composites (A) Transmission responses of separate graphene layer components. (B) Transmission characteristics of the purple composite structure and the full assembly. (C–G) Schematic diagrams of different combined structures in the graphene.

When terahertz waves are incident on the graphene-based metamaterial, the resulting transmission responses of various substructures are illustrated in Figures 2A and 2B. As illustrated in Figures 2C–2G, the blue unit represents the composition of two horizontal rectangular graphene strips, the yellow represents the central graphene unit, the cyan represents the four L-shaped graphene strips, the purple unit represents a composite unit composed of the blue and yellow units, and the green represents the overall structure of the graphene-based metamaterial. Due to their alignment with the incident electric field’s polarization, the blue and cyan units are readily excited into bright modes.

**Figure 3:**
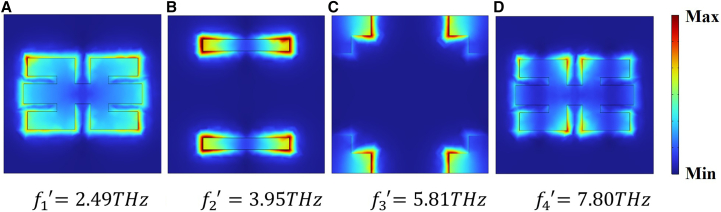
Electric field arrangements of individual units (A–D) Schematic diagrams of the electric field arrangements when each unit is excited.

The blue Lorentzian curve in Figure 2A reveals that the blue structure supports a pronounced electric dipole resonance at f^*’*^_2_ = 3.95 THz, and the electric field distribution corresponding to this resonance is presented in Figure 3B. The cyan structure is excited as a bright mode at f^*’*^_3_ = 5.81 THz. Based on this, we provide the electric field diagrams of the blue and cyan units at resonance, clearly showing that energy stored in the electric field in the cyan unit at resonance is almost entirely concentrated in the horizontal sections of the four L-shaped graphene strips, while the vertical sections exhibit only weak electric field energy, as illustrated in Figure 3C. As shown by the yellow curve in Figure 2A, the yellow unit gives rise to two dips in the transmission spectrum under incident wave excitation. The electric field energy arrangements of the yellow unit at f^*’*^_1_ = 2.49 THz and f^*’*^_4_ = 7.8 THz are given in Figures 3A and 3D, indicating that the yellow unit excites strong electric dipole resonances at frequencies f^*’*^_1_ and f^*’*^_4_.

**Figure 4:**
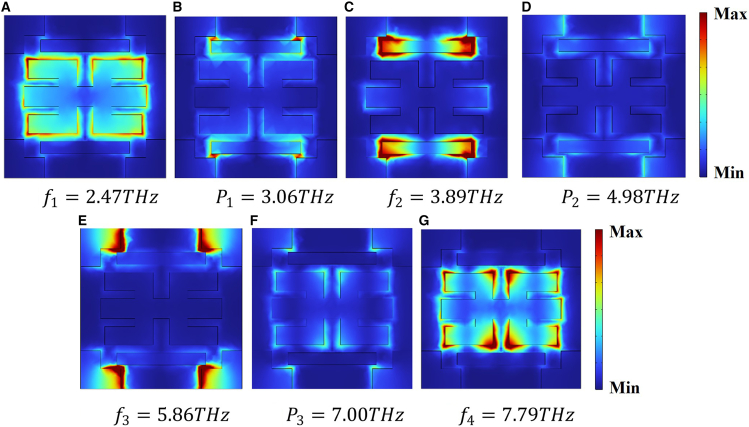
Electric field distribution at multiple resonances (A) *f*_1_ = 2.47 THz. (B) *P*_1_ = 3.06 THz. (C) *f*_2_ = 3.89 THz. (D) *P*_2_ = 4.98 THz. (E) *f*_3_ = 5.86 THz. (F) *P*_3_ = 7.00 THz. (G) *f*_4_ = 7.79 *THz*.

In Figure 2B, the purple dashed plot shows the response of the blue-and-yellow unit combination, whereas the green curve depicts the full-structure spectrum, featuring three EIT transparency peaks and four transmission dips. The three EIT transparency peaks are labeled as *P*_1_ = 3.06 THz, *P*_2_ = 4.98 THz, and *P*_3_ = 7 THz, and the four transmission dips are marked as *f*_1_, *f*_2_, *f*_3*,*_ and *f*_4_. Examining the transmission spectra of the yellow, purple, and green units at *f*_1_ = 2.47 THz reveals minimal differences among them. Figure 4A displays the electric field pattern at this frequency, with the field energy primarily localized in the yellow unit, suggesting that the transmission dip at *f*_1_ originates from the bright mode of the yellow unit. As shown by the green transmission spectrum in Figure 2B, the first EIT transparency window appears at *P*_1_ = 3.06 THz. The green and purple curves almost overlap, indicating that the purple combined unit is the main contributor to the transparency peak at*P*_1_. In this configuration, the yellow unit is excited into a bright mode, while the blue unit remains in a high-transmission state and is considered a dark mode. Energy from the electric field transfers from the yellow unit to the blue structure, causing the strong field in the yellow unit to diminish. This indicates that the transparency window is mainly caused by the near-field coupling between the blue unit and the yellow unit. In Figure 4B, the electric field layout at point *P*_1_ is shown confirming the presence of the transparency window at this location.

According to the purple curve in Figure 2B, the purple composite unit is excited into a bright mode at *f*_2_ *=* 3.89 THz, and the spatial distribution of the field at this frequency is illustrated in Figure 4C. The electric field energy concentrated in the purple composite structure indicates that the resonance dip near *f*_2_ is mainly caused by the excitation of its bright mode. The green curve in Figure 2B illustrates that the second EIT transparency window appears at *P*_2_ = 4.98 THz. Due to the near-field coupling between the purple and cyan units, the strong electric field energy originally excited in the purple composite structure disappears, resulting in the formation of the second EIT window. At this frequency, the corresponding electric field profile is depicted in Figure 4D.

Similarly, at *f*_3_ = 5.86 THz, the electric field energy mainly localizes within the cyan unit, as illustrated in Figure 4E. Thus, the transmission dip observed at *f*_3_ arises from the excitation of the bright mode in the cyan unit. As observed from the green transmission spectrum in Figure 2B, the third EIT window appears at *P*_3_ = 7 THz. Due to its weak interaction with the incoming field, the purple unit acts as a dark mode, while the cyan unit, being strongly coupled, functions as the bright mode. As a result of near-field interaction, the energy initially concentrated in the cyan unit is transferred to the purple one, resulting in the disappearance of the strong field in the cyan structure. Therefore, an EIT window emerges at *P*_3_, with its field profile illustrated in Figure 4F. The dip observed at *f*_4_ = 7.79 THz originates from the same coupling mechanism as the one at *f*_1_. In Figure 4G, the energy distribution is mainly concentrated in the yellow unit, verifying that the bright mode of this unit is responsible for the transmission dip at *f*_4_.

The abovementioned results indicate that the bright mode can be directly excited by the incident electromagnetic wave and exhibits strong radiative losses, while the dark mode is weakly radiative and cannot be directly excited by the incident wave. It can only be indirectly excited through near-field coupling with the bright mode. When the two oscillators are in a weak coupling state, destructive interference occurs between their oscillations, resulting in the appearance of a transparency window (EIT peak) in the transmission spectrum. Therefore, the previous analysis shows that the transparency windows at *P*_1_ = 3.06 THz, *P*_2_ = 4.98 THz, and *P*_3_ = 7 THz originate from the interaction among the individual units in the graphene resonators.

The adjustment of the graphene Fermi level is mainly achieved through external modulation methods such as applying gate voltage, chemical doping, and photoexcitation. In this study, the carrier concentration in graphene is altered by adjusting the back-gate or top-gate voltage, thereby enabling the dynamic tuning of its conductivity and surface plasmon resonance frequency, which results in a shift of the Fermi level. Graphene’s Fermi level is given by^37^

(Equation 7) $$\begin{array}{c} E_{f}=hv_{F}\sqrt{\left(\frac{\pi \varepsilon_{r}\varepsilon_{0}V_{g}}{ed}\right)} \end{array}$$

where *v*_*F*_ = 1 × 10^6^ m/s is the Fermi velocity, *d* denotes the distance between the graphene layer and the electrode, and *V*_g_ represents the gate voltage applied.

**Figure 5:**
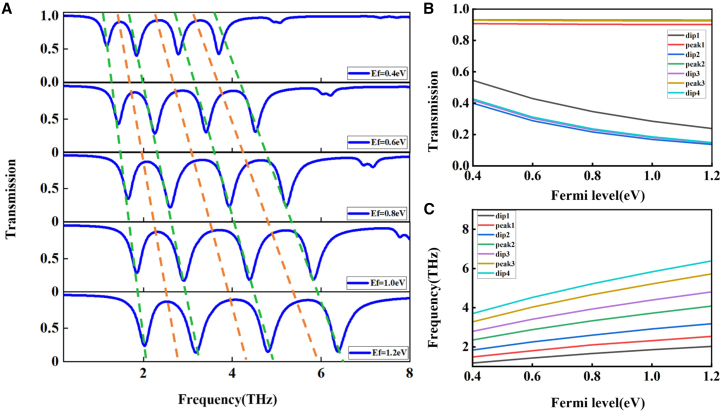
Transmission spectra under different Fermi levels (A) Transmission profiles under different Fermi levels of graphene. (B) Transmission intensity at resonance versus Fermi level. (C) Resonant frequency shift under Fermi level modulation.

Figure 5A illustrates that increasing the Fermi level causes the transmission curve to shift toward higher frequencies. This shift indicates that a greater carrier density strengthens the coupling in surface plasmon resonances. Figure 5B depicts the transmittance changes occurring at the resonance frequencies. As the Fermi level increases, the transmittance at the dip points (dip1 to dip4) continuously decreases, while the transmittance at the peak points (peak1 to peak3) remains almost unchanged, suggesting that the maximum transparency of the transparency windows is not affected by changes in the Fermi level. Figure 5C indicates that increasing the Fermi level results in a shift of the resonance frequency toward higher values.

**Figure 6:**
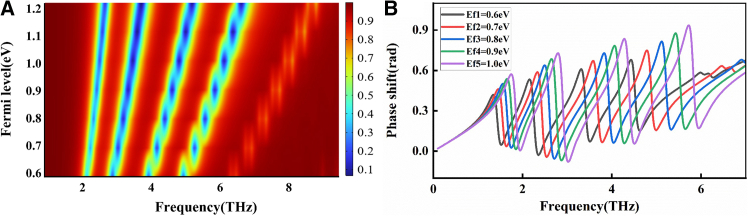
3D transmission spectrum variations and phase shifts (A) 3D illustration of transmission spectrum variations induced by changes in Fermi level. (B) Phase shift corresponding to various graphene Fermi levels.

Figure 6A further confirms the trend that the characteristic frequency points corresponding to different Fermi levels shift regularly along the frequency axis. Further analysis reveals that near the frequency point *P*_2_ = 4.98 THz, the dip depth within the transmission curve consistently declines as the Fermi level is raised, while the transmission peak remains relatively stable. This leads to an expansion of the effective bandwidth of the EIT window, which remains consistently greater than that of conventional narrowband structures. A similar phenomenon is also observed at the first and third transparency windows. As shown in Figure 6B, the phase shift curves corresponding to different Fermi levels exhibit a pronounced negative jump at the resonance frequency, which results from the strong dispersion within the transparency window. This phenomenon leads to a significant reduction in the group refractive index and provides a new design approach for phase encoders.

## Results

### Quad-band optical switch

**Figure 7:**
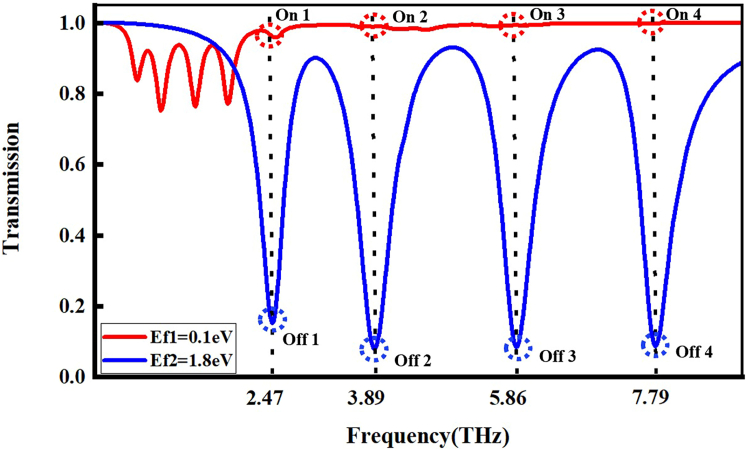
Graphene-based metamaterial realizes four-frequency optical switching at 2.47, 3.89, 5.86, and 7.79 THz

The terahertz optical switching function of the graphene-based metamaterial originates from the shift of the transmission curve toward the blue end of the electromagnetic spectrum due to Fermi level tuning. As shown in Figure 7, the transparency peaks and transmission dips in the transmission spectrum correspond to the “ON” state (high transmission) and the “OFF” state (low transmission) of the device, respectively. With the Fermi level tuned from 0.1 up to 1.8 eV, the transmittance at the frequency point of 2.47 THz switches from a peak value (ON state) to a dip value (OFF state). Conversely, reducing the Fermi level from 1.8 down to 0.1 eV reverses the switch from the OFF state to the ON state.

In a similar manner, when the Fermi level is maintained at 0.1 eV, the transmission spectrum shows elevated transmittance at 3.89, 5.86, and 7.79 THz, indicating the “ON” state. Upon raising the Fermi level to 1.8 eV, the transmission at these frequencies transitions from peaks to dips, corresponding to the “OFF” state. By using dual Fermi level modulation at 0.1 and 1.8 eV, dynamic and reversible switching can be achieved at four characteristic frequencies, enabling the construction of a four-channel optical switching system operating across multiple frequency bands. Key parameters such as extinction ratio (ER), insertion loss (IL), and MD serve as critical metrics for evaluating the optical switch’s performance, with the specific calculation formulas given as follows^38^^,^^39^^,^^40^

(Equation 8) $$\begin{array}{c} MD=\frac{\left|T_{on}-T_{off}\right|}{T_{on}}\times 100\% \end{array}$$

(Equation 9) $$\begin{array}{c} IL=-10\;\log_{10}T_{on}\left(dB\right) \end{array}$$

(Equation 10) $$\begin{array}{c} ER=10\;\log_{10}\left(T_{on}/T_{off}\right)\text{.} \end{array}$$

here, *T*_*on*_ indicates the transmission when the switch is turned “ON”, while *T*_*off*_ corresponds to the transmission when it is turned “OFF.” At 2.47, 3.89, 5.86, and 7.79 THz, the quad-band optical switch achieves MDs of 84.2%, 92.1%, 91.5%, and 91.3%, respectively. The associated ILs at these frequencies are 0.17, 0.05, 0.03, and 0.01 dB, while the ERs reach 8, 11.0, 10.7, and 10.6 dB in sequence. The designed graphene-based metamaterial device achieves a MD above 84.2% at all four characteristic frequencies (reaching up to 92.1%), with IL controlled below 0.17 dB (as low as 0.01 dB), and ER consistently greater than 8 dB (up to 11 dB). According to Table 1, relative to previously reported graphene-based optical switches, the proposed device demonstrates enhanced performance across crucial indicators such as MD and IL. Its wideband, multi-channel response capability offers an innovative solution for implementing terahertz multi-frequency multiplexed optoelectronic systems.

**Table 1:** Comparison of optical-switching performance between this work and previous reports

Reference	Modulation mode	Min MD	Max MD	Min IL	Max ER
Guo et al.^41^	triple frequency	44.0%	80.6%	–	–
Duan et al.^42^	dual frequency	46.6%	85.1%	–	–
Meng et al.^43^	five frequency	70.5%	87.1%	–	8.9 dB
Xu et al.^44^	five frequency	79.8%	87.5%	0.05 dB	9 dB
Zhou et al.^45^	five frequency	76.7%	88.1%	–	–
Li et al.^46^	six frequencies	70.1%	88.5%	0.15 dB	–
This work	quad frequency	84.2%	92.1%	0.01 dB	11 dB

### Three-bit encoder

By independently tuning the Fermi levels of the yellow, cyan, and blue components within the graphene architecture, the frequency shift of the EIT window can be effectively controlled, thereby modifying the transmittance amplitudes at 3.18, 4.8, and 6.38 THz. This feature enables the device to realize three-bit encoding functionality. In this work, logical states are defined based on transmittance amplitude, using a threshold of 50% transmittance: a transmittance below the threshold represents logic “0,” while a transmittance above the threshold represents logic “1.”

**Figure 8:**
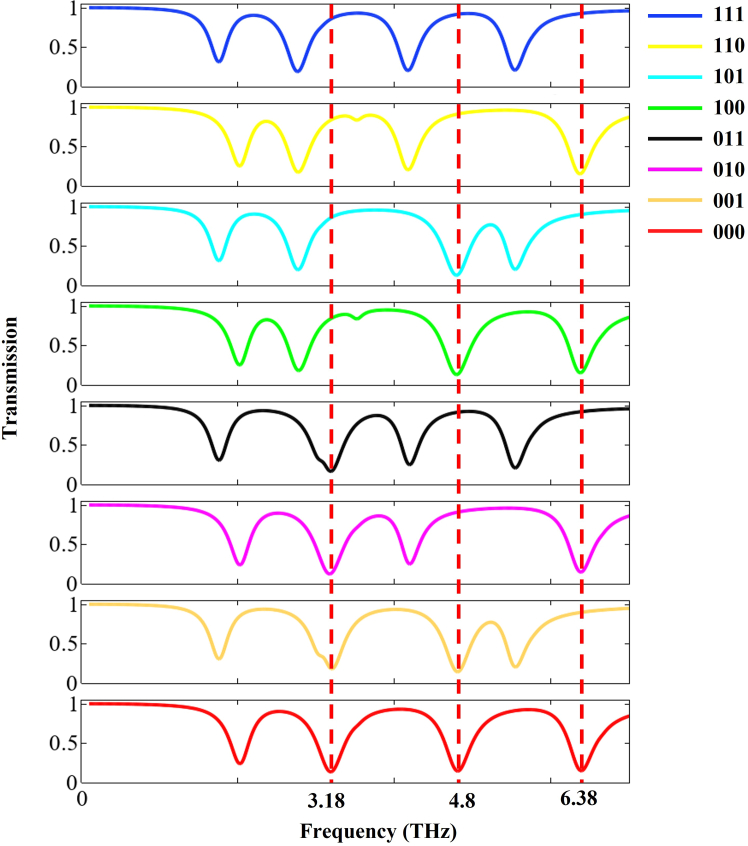
Schematic diagram of the three-bit encoder operating under *x*-polarized state

Under *x*-polarized terahertz wave normal incidence, eight coding modes are regulated through the combination of Fermi levels among the unit cells. Figure 8 presents the three-bit coding map under *x*-polarization. The eight subfigures within the diagram fully illustrate the switching process of logical states from “111” to “000.” When the Fermi levels of the blue, yellow, and green units are all set to 0.9 eV, the transmittances at 3.18, 4.8, and 6.38 THz are all above the set threshold, corresponding to the logical state “111.” If only the Fermi level of the yellow unit is increased to 1.2 eV, a blueshift of the transmission dip at 6.38 THz causes the transmittance to fall below the threshold, while the transmittances at 3.18 and 4.8 THz remain above the threshold, corresponding to the logical state “110.”

Similarly, if only the cyan unit’s Fermi level is elevated to 1.2 eV, the logical state corresponds to “101.” When both the yellow and cyan units have their Fermi levels increased to 1.2 eV and the blue unit remains at 0.9 eV, it corresponds to the logical state “100.” If only the blue unit’s Fermi level is raised to 1.2 eV while the yellow and cyan units remain at 0.9 eV, it corresponds to “011.” When the blue and yellow units are both increased to 1.2 eV and the cyan unit remains at 0.9 eV, the corresponding logical state is “010.” If the blue and cyan units are increased to 1.2 eV while the yellow unit remains at 0.9 eV, this corresponds to “001.” When all three Fermi levels are raised to 1.2 eV, the transmittances at all three frequency points fall below the threshold, corresponding to the logical state “000.” When the transmission of each channel is assigned as “1” for high and “0” for low, all eight possible combinations of binary states (000–111) can be realized, as summarized in Table 2. The encoder demonstrates strong performance, achieving a peak MD of 84%, an ER as high as 8 dB, and IL as low as 0.33 dB, thereby presenting a novel approach to designing graphene-based metamaterial encoders.

**Table 2:** Mapping between graphene Fermi levels, transmission states, and 3-bit binary codes

State no.	Fermi level of yellow units	Fermi level of cyan units	Fermi level of blue units	Transmission (3.18/4.8/6.38 THz)	Binary code
1	1.2 eV	1.2 eV	1.2 eV	0 0 0	000
2	1.2 eV	1.2 eV	0.9 eV	0 0 1	001
3	1.2 eV	0.9 eV	1.2 eV	0 1 0	010
4	1.2 eV	0.9 eV	0.9 eV	0 1 1	011
5	0.9 eV	1.2 eV	1.2 eV	1 0 0	100
6	0.9 eV	1.2 eV	0.9 eV	1 0 1	101
7	0.9 eV	0.9 eV	1.2 eV	1 1 0	110
8	0.9 eV	0.9 eV	0.9 eV	1 1 1	111

### Slow light device

The graphene-based metamaterial proposed in this work demonstrates remarkable slow-light properties, attributed to the pronounced dispersion caused by the rapid phase transition occurring at the EIT resonance. The strength of this slow-light phenomenon is evaluated using the group refractive index, defined by the following equation^47^

(Equation 11) $$\begin{array}{c} n_{g}=\frac{C}{d}\times \tau_{g}=-\frac{C}{d}\times \frac{d\varphi \left(\omega \right)}{d\omega }\text{.} \end{array}$$

**Figure 9:**
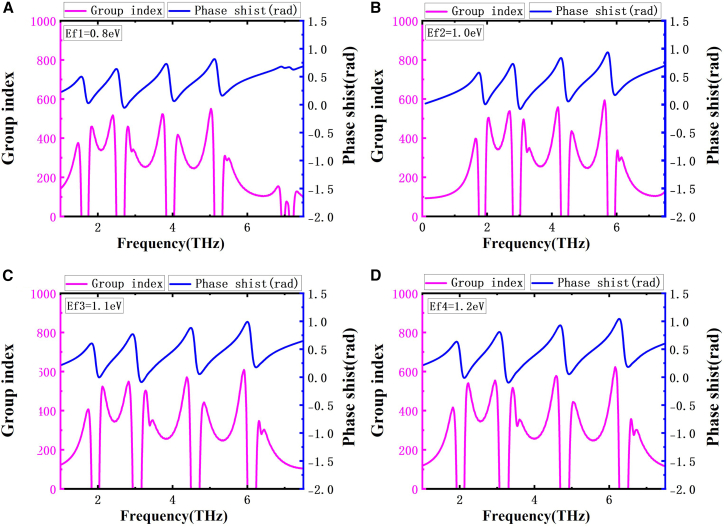
Phase shift and group index at different Fermi levels (A–D) Phase shift and group refractive index at Fermi levels of 0.8, 1.0, 1.1, and 1.2 eV. Blue and purple curves represent phase shift and group index, respectively.

In its calculation formula, *C* = 2.998 × 10^8^ m/s indicates the vacuum light speed, *d* stands for the substrate thickness, and *φ*(*ω*) describes the phase shift of transmission. Figures 9A–9D illustrate how the phase and the associated group refractive index vary with different Fermi levels. From Figure 9A, one can observe that near the EIT transmission window, the transmission phase undergoes an abrupt jump accompanied by strong dispersion. Since the group refractive index is proportional to the rate of change of the phase with respect to frequency, a more abrupt phase variation leads to a higher group refractive index. Figure 9A demonstrates that a Fermi level of 0.8 eV corresponds to a peak group refractive index of 548; and as shown in Figure 9D, with the Fermi level elevated to 1.2 eV, the group refractive index of the device significantly increases, reaching a peak value of 623. This phenomenon indicates the slow-light effect is dynamically tunable through Fermi level control. This characteristic offers an innovative solution for developing new tunable optical delay devices and holds significant application prospects in terahertz communication systems, optical quantum storage, and on-chip signal processing.

### Incident wave angle-insensitive device

**Figure 10:**
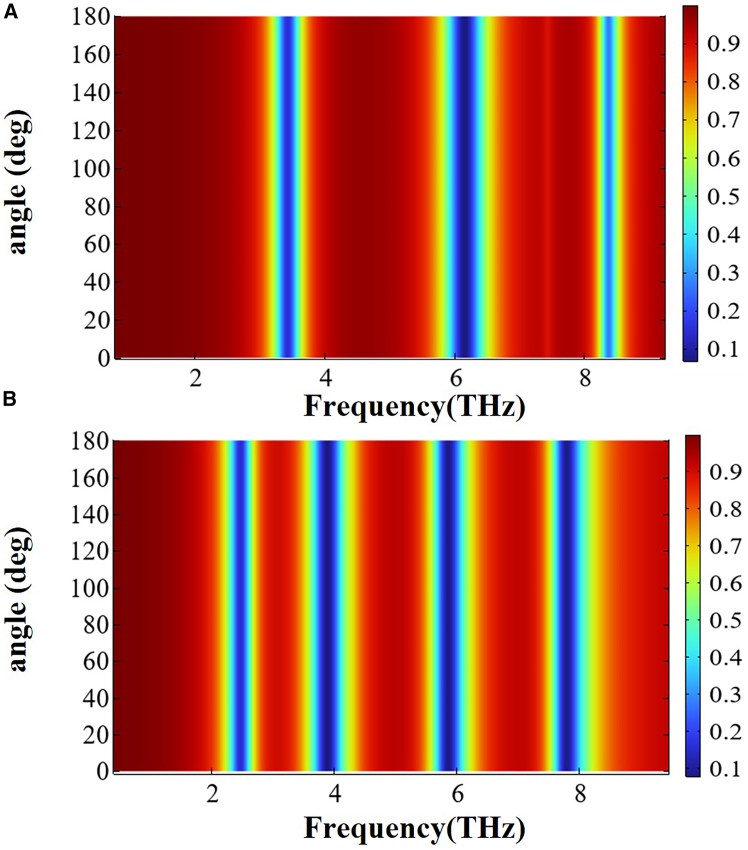
3D transmission spectra under varying polarization and incidence angles (A and B) 3D transmission spectra under *x*- and *y*-polarized incidence at varying angles.

To examine how variations in the incident wave angle affect device performance, this study analyzed the transmission spectral responses over an incident angle range from 0° to 180° under *x*- and *y*-polarized light. Figures 10A and 10B displays 3D transmission maps versus incident wave angle for the two polarization states. Figure 10 demonstrates that, under *x*- and *y*-polarized incidence conditions, the intensity distribution and resonance frequency shifts of the transmission spectra both exhibit high consistency, demonstrating excellent angle-insensitive characteristics. This angle insensitivity arises from the high symmetry of the structure, which enables any incident polarization direction to excite equivalent resonant modes. The results indicate that this feature removes the stringent polarization-alignment requirements of conventional metamaterials, providing an innovative solution to reduce the complexity of optical systems.

## Discussion

This study proposes a graphene-based triple-band EIT metamaterial device. Under normal incidence of *x*-polarized terahertz waves, the structure exhibits three EIT transparency windows with transmission rates exceeding 90% and four transmission dips. Adjusting the graphene Fermi level causes the resonance peaks in the transmission spectrum to shift in frequency. Utilizing this phenomenon, a quad-band optical switch can be achieved under *x*-polarized terahertz wave incidence, featuring strong MD, minimal IL, and a high ER. The device achieves a MD as high as 92.1% is attained, alongside an IL of only 0.01 dB and an ER of 11 dB. The designed system can also achieve triple-bit encoder functionality at 3.18, 4.8, and 6.38 THz, achieving 84% peak MD while maintaining 8 dB maximal ER and 0.33 dB minimal IL. In addition, the device enables dynamic tuning of the slow-light effect, with a maximum group index of 623. This study further probes the transmission spectra dependence on *x*- and *y*-polarized terahertz waves over 0°–180° incident angles, revealing that the designed structure demonstrates exceptional wide-angle insensitivity. Therefore, the designed metamaterial structure integrates multifunctional capabilities, including multi-band optical switching, ternary coding, and tunable slow-light effects, demonstrating great potential for applications in terahertz high-speed communication, real-time imaging and sensing, on-chip photonic encoding and computing, as well as other related fields.

### Limitations of the study

This protocol is constrained by assumptions inherent in numerical simulations and material models. Finite-element calculations typically assume perfect periodicity and lossless substrates, which may differ from realistic material losses. In addition, numerical dispersion and mesh-dependent errors can slightly shift the resonance frequencies, necessitating careful mesh refinement and solver-convergence checks.

## Resource availability

### Lead contact

Further information and requests for resources and reagents should be directed to and will be fulfilled by the lead contact, Jun Zhu (zhujun1985@gxnu.edu.cn).

### Materials availability

This study did not generate new materials.

### Data and code availability

• This study did not generate or analyze any datasets.
• The simulation scripts used in this study are available from the lead contact upon reasonable request.
• Any additional information required to reanalyze the simulations reported in this paper is available from the lead contact upon request.

## Acknowledgments

We acknowledge support from the Guangxi Natural Science Foundation (2023GXNSFAA026015) and the 10.13039/501100001809National Natural Science Foundation of China (grant no. 51965007).

## Author contributions

J.D. conducted experiments and wrote the manuscript under the supervision of Z.J. All authors contributed to the draft and prepared the final version.

## Declaration of interests

The authors declare no competing interests.

## STAR★Methods

### Key resources table

**Table:** 

REAGENT or RESOURCE	SOURCE	IDENTIFIER
COMSOL Multiphysics	COMSOL, Inc.	Version 6.2
MATLAB	MathWorks	R2021a
Windows 10	Microsoft	https://www.microsoft.com/en-us/software-download/windows10
Accession codes	-	N/A

**Other**		

CPU laptop	Intel	Intel(R) Core(TM) i5-9400F processor (2.90 GHz)

### Method details

#### Before you begin

In COMSOL Multiphysics, a unit-cell model of the graphene-based resonant structure is first constructed, and the electromagnetic field distribution is analyzed using the finite element method (FEM). Floquet periodic boundary conditions are applied along the x- and y-directions to simulate an infinite periodic array, while terahertz waves are set to propagate normally along the z-direction with selectable x- or y-polarization.

During the simulation, solver parameters must be properly configured, including frequency-domain settings and mesh refinement, to ensure accurate calculation of the electromagnetic field distribution.

This protocol covers the key computational details when using the wave optics module, including the boundary conditions, initial conditions, and main physical parameters used in the simulation, providing complete preparation for subsequent analyses.

#### Installation of system resources and applications

The computational workflow was performed on a Windows 10 Professional workstation equipped with an Intel(R) Core(TM) i5-9400F processor (2.90 GHz) and an NVIDIA GeForce GTX 1650 graphics card. COMSOL Multiphysics and the Wave Optics Module were installed and configured with the required solver settings, material libraries, and meshing tools. After the environment setup, all electromagnetic simulations were carried out using the finite element method.

#### Setting of metamaterial structure

A graphene-based metamaterial unit-cell model was constructed in COMSOL, where the graphene layer was implemented as part of a composite resonator. To emulate an infinitely periodic array, Floquet periodic boundary conditions were applied along the x- and y-directions. A normally incident terahertz wave was introduced along the z-axis, and adaptive mesh refinement was applied to ensure numerical accuracy.

#### Structural transmission spectrum analysis

The transmission characteristics of the metamaterial were computed using the frequency-domain solver. An x-polarized plane wave was launched from the incident port along the z-direction, and S-parameters were extracted at the output port to obtain the transmission spectrum. Resonant dips and electromagnetically induced transparency (EIT) peaks were identified from the frequency-dependent curves, and their evolution under different graphene Fermi levels was analyzed to evaluate optical switching, multiband modulation, and encoding functionality.

#### Electric field response analysis

The electric-field distributions were computed using FEM-based simulations under the same periodic boundary conditions. Field maps at selected frequencies were extracted to examine resonance formation, modal hybridization, and coupling interactions among the rectangular, L-shaped, and trident-shaped resonators. Variations in field localization and EIT behavior under different graphene Fermi levels provided insights into optimizing the metamaterial for enhanced transmission performance and modulation depth.

### Quantification and statistical analysis

All numerical results were obtained from finite-element simulations performed in COMSOL Multiphysics. The eigenfrequency and frequency-domain studies were solved using the frequency-dependent material models described in the STAR Methods section. Mesh convergence was verified by progressively refining the mesh until variations in the resonance frequency and field distributions were below 0.5%.

The accuracy of the eigenmodes was validated by confirming the continuity of dispersion curves and the absence of numerical artifacts. Field intensities and band diagrams were post-processed using COMSOL and MATLAB without further statistical treatment, as the results are deterministic solutions of Maxwell’s equations.

#### Expected outcomes

Execution of this protocol will reproduce the transmission characteristics of the graphene-based EIT metamaterial, including three high-transmittance EIT windows and four distinct resonance dips under normal incidence of x-polarized terahertz waves. Users will observe frequency-tunable resonance peaks by adjusting the graphene Fermi level, enabling a stable quad-band optical switching response with a modulation depth up to 92.1%, an insertion loss as low as 0.01 dB, and an extinction ratio of 11 dB.

The protocol will also yield reproducible ternary-bit encoding states at 3.18, 4.8, and 6.38 THz, where the device maintains an 84% modulation depth, 0.33 dB minimum insertion loss, and up to 8 dB extinction ratio. Slow-light analysis will show a dynamically tunable dispersion response, with the group index reaching values as high as 623.

Additionally, users can expect to confirm the structure’s wide-angle insensitivity by examining transmission responses under x- and y-polarized terahertz waves across incident angles from 0° to 180°. Overall, the protocol supports consistent reproduction of the device’s multifunctional performance, including multi-band optical switching, multi-level encoding, and tunable slow-light effects.
